# Catarrhal Phthisis in a Cow

**Published:** 1881-10

**Authors:** George H. Parkinson

**Affiliations:** Middletown, Conn.


### VII.—CATARRHAL PHTHISIS IN A COW.

EDITOR OF JOURNAL OF COMPARATIVE MEDICINE:

The cow from which the lungs were removed was three-quarter Jersey and one-quarter Ayrshire, aged eight. The animal had been for four years on the same farm. Up to March, 1881, the cow had been in the best of health. In March, however, she commenced coughing, and lost flesh rapidly until her death in July. The animal was examined by a so-called veterinary surgeon, and he diagnosticated consumption. At the necropsy the lungs were the principal organs diseased. A portion of them was consolidated and gray in color. The lungs were examined microscopically, but no evidence of miliary tubercles found. But the condition commonly known as catarrhal phthisis existed; that is, large portions of the lung were consolidated, the alveoli being filled with degenerating epithelum and debris, or cheesy masses. The case is of interest, I think, in showing how rapidly the disease progressed and terminated in the death of the animal. Would the milk of such an animal be injurious?

GEORGE H. PARKINSON, M.D.

MIDDLETOWN, Conn.

